# Investigating the Antagonistic Effect of Indigenous Probiotics on Carbapenem-Resistant *Pseudomonas aeruginosa* Strains

**DOI:** 10.1155/2023/6645657

**Published:** 2023-09-28

**Authors:** Azita Ghiaei, Seyed Mahdi Ghasemi, Dariush Shokri

**Affiliations:** ^1^Department of Biotechnology, Faculty of Biological Sciences and Technology, Shahid Ashrafi Esfahani University, Isfahan, Iran; ^2^Nosocomial Infection Research Center, Isfahan University of Medical Sciences, Isfahan, Iran

## Abstract

**Introduction:**

With the increase of hospital infections due to the indiscriminate use of antibiotics, multidrug resistance has increased, decreasing the effectiveness of antibiotics against these infections. For this reason, the identification of alternative agents such as probiotics has been considered. The aim of this study was to isolate and identify effective probiotics against carbapenem-resistant *Pseudomonas aeruginosa* strains. *Material and Methods*. During a period of eight months, isolates of *P. aeruginosa* were collected from patients in three hospitals in Isfahan. The presence of metallo-beta-lactamase enzymes was determined by the combination disc test (CDT). The inhibitory and antimicrobial activities of 20 probiotic bacteria isolated from local dairy products against these strains were investigated by agar dilution. Two probiotic strains that showed broader inhibition results were selected, and the values of the lowest inhibitory concentration (MIC) and the lowest lethal concentration (MBC) and their antibiofilm effect were determined using the microtiter plate method. The concentration of organic acids was done by HPLC. *Findings*. Of the 100 samples isolated and identified, 61 samples (61%) exhibited multiple drug resistance (MDR) and were selected for further investigation. Phenotypic diagnosis of the presence of metallo-beta-lactamase enzymes revealed that 74.5% of the strains were positive. The results showed that these two probiotics killed *P. aeruginosa* strains after only one hour, and the inhibition mechanism was due to the presence of lactic acid and acetic acid. The antibiofilm effect of these two probiotics was at concentrations of 1/2 and 1/4.

**Conclusion:**

The two Lactobacillus isolates had potential antimicrobial and antibiofilm properties against all carbapenem-resistant *P. aeruginosa* strains, even at thinner dilutions. Considering the broad activity of this strain, it can potentially be used for biocontrol of these strains.

## 1. Introduction

As a global issue that is getting worse, antibiotic resistance drives widespread research into natural, harmless antibiotic alternatives like probiotics [1]. The use of probiotics, particularly lactobacilli, as a safe and natural living countermeasure to antibiotic-resistant and food-spoilage microbes has recently been given another look as an alternative to antibiotics and some chemical preservatives [2]. By inhibiting pathogen adhesion through competition, enhancing the host's immune system, and producing inhibitory substances including organic acids, hydrogen peroxide, and proteinaceous compounds (such as bacteriocins and antibacterial peptides), lactobacilli, which are mostly probiotic organisms, can operate as microbial barriers against infections [3, 4].

Infections in nosocomial settings are frequently brought on by *Pseudomonas aeruginosa* [5, 6]. The ability of *P. aeruginosa* to acquire and spread resistance both vertically and horizontally in the hospital environment and its propensity to persist on both animate and inanimate things around the patient, including antiseptic solutions, are all attributed to its metabolic inventiveness [7]. Multi-drug-resistant (MDR) bacteria are on the rise and posing a serious health risk to people all over the world [8]. Hospital-acquired infections brought on by MDR bacteria have increased mortality, morbidity, and treatment costs while also putting patients' lives in threat [9]. The rise in MDR bacteria and restrictions on antibiotic use because of its negative effects have prompted researchers to look into potential substitutes [10, 11].

When treating severe infections brought on by MDR Gram-negative bacilli, carbapenems which were first introduced in the 1980s are the last line of defense [12]. In *P. aeruginosa*, resistance to carbapenem is brought on by altered penicillin-binding proteins, enhanced efflux system, decreased outer membrane permeability, and carbapenemases, an enzyme that hydrolyzes carbapenem [13]. *P. aeruginosa* that produces MBL is a growing hazard and a source of concern because it has become one of the most feared resistance mechanisms [14, 15]. Their large substrate range, propensity for horizontal transfer, and lack of inhibition by serine *β*-lactamase inhibitors such clavulanic acid, sulbactam, and tazobactam set them apart from other carbapenemases [16].

The biofilm, a complex collection of bacteria encased in an extracellular polymeric substance (EPS) matrix, is one of the most important mechanisms for a species' survival when its environment unexpectedly changes, such as when the availability of nutrients or when the temperature changes [17]. *P. aeruginosa* is a well-known producer of biofilms, making it a great model to investigate the process. For *P. aeruginosa* to compete, live, and dominate in the polymicrobial environment of the cystic fibrosis lung, a strong biofilm is an essential tool [18–20]. Probiotic administration has been found to be useful in preventing and/or combating the biofilm-related infection. The purpose of this study was to isolate and identify effective probiotics against carbapenem-resistant *P. aeruginosa* strains.

## 2. Material and Methods

### 2.1. Sample Collection and Identification

This cross-sectional descriptive study was done on different strains of *P. aeruginosa* isolated from clinical samples including urine, respiratory, cerebrospinal fluid (CSF), abdominal fluid, pericardial fluid, and blood. The samples were collected from patients hospitalized in three hospitals in the province of Isfahan (Amin, Asgariye, and Milad), as well as collected in the private laboratory of Noble Isfahan. These samples were incubated at 37°C for 24 hours while being cultivated on blood agar medium and eosin methylene blue (EMB) media (HiMedia Company, India). The pure strains were recognized using biochemical testing and Gram staining [21].

### 2.2. Antibiotic Susceptibility Testing

Antibiotic resistance patterns of clinical isolates were determined using disc diffusion method according to the CLSI guidelines [22]. The following antibiotics were used: levofloxacin (5 *μ*g), ciprofloxacin (5 *μ*g), meropenem (10 *μ*g), imipenem (10 *μ*g), piperacillin-tazobactam (100-10 *μ*g), amikacin (30 *μ*g), gentamicin (10 *μ*g), ampicillin sulbactam (10 *μ*g), ceftazidime (30 *μ*g), and cefepime (30 *μ*g) (BD, USA). *P. aeruginosa* ATCC 27853 was used as reference strain.

### 2.3. Combination Disc Test (CDT)

On MH agar, a lawn culture of the test isolate was performed (0.5 McFarland opacity standard). On inoculated plates, two imipenem discs weighing 10 *μ*g each were put. 10 *μ*L of a 0.5 M EDTA solution was added to one of the imipenem discs. If the zone of inhibition of imipenem+EDTA discs compared to imipenem alone is >7 mm after overnight incubation, the test was judged positive [23].

### 2.4. Isolation of *Lactobacillus* Strains

The search for probiotic bacteria from different native sources including yogurt and milk was done. Samples were collected from different regions of Isfahan province in Iran including Shahreza, Golpayegan, Khorasgan, and Najafabad. For the purpose of isolating bacteria, 1 mL of each dairy sample was homogenized, suspended in a solution of 2% *w*/*v* sodium citrate from the Merck Company in Germany, and added to 10 mL of MRS broth from the HiMedia Company in India. The samples were then incubated for 24 hours at 37°C. 0.02 mL of the solutions was distributed for 48 hours on MRS agar media following incubation. Catalase testing, Gram staining, and biochemical tests such as growth at 15 and 45°C, acid and gas production from glucose, NH3 production from arginine, and sugar fermentation including arabinose, cellobiose, mannitol, mannose, melebiose, raffinose, ribose, salicin, rhamnose, and xylose were used to identify the strains [24].

### 2.5. Assessment of Antibacterial Activity of *Lactobacillus* Strains

Detection of antibacterial activity of *Lactobacillus* strains was performed using the following methods: agar well diffusion method and MIC/MBC test were investigated.

### 2.6. Agar Well Diffusion Method

The cell-free supernatant of *Lactobacillus* cultures was collected and used in agar well diffusion method as previously described [25]. The plates were incubated 24 h at 37°C, and then, antibacterial activity was recorded as growth-free inhibition zones around the wells. After incubation period, the growth inhibition zone was measured and compared with that of the control group.

### 2.7. Broth Microdilution Assay

According to earlier descriptions, the antibacterial activity (MIC and MBC) of cell-free supernatants of probiotics growing in the presence or absence of probiotics against clinical isolates of MDR *P. aeruginosa* was measured. No more turbidity was produced after that to verify correctness. It was then grown on blood agar medium and incubated once more for 24 hours at 37°C, which means that probiotics have an inhibiting effect on the pathogenic strain [26].

### 2.8. Time-Kill Test in Cocultures

This test was carried out in order to determine the desired probiotic after a minimum period of time. The time-kill assay was performed by coculture of the *P. aeruginosa* cells and cell-free supernatant of *Lactobacillus* spp. A suspension equivalent to 0.5 McFarland turbidity was prepared from the *P. aeruginosa* strain. Then, the supernatant to suspension was added. Then in zero, 1 hour, 2 hours, 4 hours, 8 hours, 12 hours, 24 hours, and 48 hours, it was cultured on blood agar medium and put in an incubator at 37°C for 24 hours [27].

### 2.9. High-Performance Liquid Chromatography (HPLC)

The detected *Lactobacillus* strains were grown for 72 hours in MRS broth medium before being centrifuged for 10 minutes at 10,000 g. The bacterial pellet's supernatant was taken out, and it was then filtered through a 0.25 m syringe filter. To ensure the filtrate's sterility (no lactobacilli growth), it was then recultured for 72 hours in MRS broth medium. The filtrate was fed into the HPLC apparatus in a volume of 20 microliters. On reversed-phase HPLC columns C18 (25 cm 4.6 mm), chromatographic separation was accomplished using an aqueous mobile phase (phosphate buffer-CH3CN 10 mM, at a flow rate of 1 mL·min^−1^, pH 3.6, and UV absorbance was measured at 282 nm at room temperature at 25°C [25]).

### 2.10. Bile Salts and Low pH Tolerance Tests

According to Haghshenas et al., high bile salt concentrations and low pH tolerance were examined in *Lactobacillus* strains that showed the best antibacterial activity [28]. The strains were incubated for 24 hours at 37°C in 5 mL of MRS growth medium. Bacterial cells were then centrifuged at 2000 g for 15 min, before being thoroughly cleaned twice with PBS (phosphate-buffered saline). The cells were then resuspended in two media: thioglycollate broth medium, which was made by combining MRS broth with 0.3% (*w*/*v*) ox gall and 0.2% (*w*/*v*) sodium thioglycollate, and 1 mL of PBS with a pH of 2.5. For three hours, these media were incubated at 37°C. The cell survival rate was calculated using the pour plate method and compared to the rates of cell survival of strains cultured in PBS for 0 and 3 hours. After creating the highest serial dilution, the *Lactobacillus* strains were cultured in MRS broth medium at 37°C for 48 hours. In order to calculate the survival rates, single colony counting was used. The mean values for each bacterial isolate were taken into consideration after this experiment was conducted three times.

### 2.11. Antibiofilm Effect of Lactobacilli

Using the method of Ma et al., biofilm production by *P. aeruginosa* strains was evaluated [29]. For 24 hours, these strains were raised at 37°C in tryptic soy broth (TSB) that included 0.25% glucose. The cultures were diluted (1 : 100) in TSB medium before being added to 200 *μ*L of the bacterial suspension on sterile 96-well polystyrene microtiter plates. The plates were then incubated for 24 hours at 37°C. The wells were cleaned three times in 300 *μ*L of distilled water, dried, and dyed for 15 minutes in 200 *μ*L of 0.1% crystal violet (Merck Company, Germany). The wells were stained with 200 *μ*L of 30% acetic acid (Merck Company, Germany) in water after being cleaned three times with distilled water. Using an ELISA reader (Stat Fax-2100), the absorbance in the destaining solution was determined at 570 nm. According to the approach outlined above, lactobacilli were also grown on their own to investigate the production of monospecies biofilms. However, in this case, the lactobacilli were diluted one hundredfold in MRS medium that contained 0.2% sucrose. Using pH test strips, the biofilm cultures' final pH was determined. *P. aeruginosa* strains obtained using the aforementioned approach were added to 100 *μ*L of 0.5 McFarland standard native cell-free supernatant of lactobacilli in MRS medium, and 100 *μ*L of *P. aeruginosa* strains was prepared using the aforementioned method simultaneously to 96-well-connected microtiter plates. This was done to investigate the antibiofilm production of *P. aeruginosa* strains by lactobacilli. *P. aeruginosa* strains were initially allowed to grow in wells for 24 hours and create biofilms; then, cell-free supernatant of *Lactobacillus* strains was added to the wells and was incubated for 24 hours at 34°C to evaluate the removal of biofilms formed by *P. aeruginosa* strains using *Lactobacillus* strains. Using the procedure outlined above, the amount of biofilm that was reduced as a result of the cell-free *Lactobacillus* supernatant's antibiofilm impact was measured. The samples were categorized as strong (4x ODc < ODi), moderate (2x ODc < ODi 4x ODc), weak (ODc < ODi < 2x ODc), or nonbiofilm producers (ODi < ODc) based on their optical densities (ODi) and the average OD of the negative control (ODc). Each test was run in triplicate, with *P. aeruginosa* ATCC 27853 serving as the positive control and no infected medium serving as the negative control.

### 2.12. MTT Test to Investigate the Cytotoxic Effect of Probiotics on L929 Fibroblast Cell Line

The 3-(4,5-dimethylthiazol-2-yl)-2,5-diphenyltetrazolium bromide (MTT) test was used to assess cytotoxicity (Sigma-Aldrich, St. Louis, MO, United States).

Normal subcutaneous connective tissue (L929) cell lines were purchased from Pasteur Institute (Tehran, Iran). In brief, these cells were cultured overnight at 37°C in a 5% CO_2_ atmosphere in Dulbecco's modified Eagle medium (DMEM) supplemented with 10% fetal bovine serum (FBS). Approximately 5 × 10^5^ cells were seeded per well in 96-well microplates. Once the cells had adhered to the bottom of the wells as monolayers, 100 *μ*L of five different dilutions (1, 0.5, 0.25, 0.125, and 0.062) of each cell-free supernatant were added to their respective wells. The microplates were then incubated at 37°C for 48 hours. Following the incubation period, 50 *μ*L of the MTT reagent (at a concentration of 004/0 g/mL in sterile PBS) was added to each well, and the microplates were further incubated at 37°C for an additional four (4) hours. Subsequently, the culture medium in the wells was aspirated, and 50 *μ*L of dimethyl sulfoxide (DMSO) from Merck, Germany, was added to dissolve the formazan crystals. The results were measured by recording the absorbance at 540 nm using a microplate reader (BioTek Instruments, Inc., Vermont, USA) [30, 31].

### 2.13. Identification of Selected Lactobacilli

The best *Lactobacillus* strains with inhibitory and antibiofilm properties against pathogenic strains were first identified using previously established conventional methods based on their morphological, cultural, and biochemical characteristics [25]. The identified bacteria with these classical tests were confirmed by 16S ribosomal DNA (rDNA) PCR method with the universal primers including universal 1 (27f): AGAGTTTGATCCTGGCTCAG and universal 2 (1492r): TACGGYTACCTTGTTACGACTT (Cinnagen Company, Iran). The amplification of 16S rDNA gene was as follows: 1 cycle of initial denaturation (94°C for 5 min), 30 cycles (denaturation in 94°C for 30 s, 54°C as annealing temperature for 30 s, extension in 72°C for 5 min), and 1 cycle of final extension in 72°C for 5 min. At Macrogen, Inc. (Seoul, Korea), direct sequencing was used to identify the nucleotide sequences of positive PCR products. Sequences were matched to BLAST search results from the National Center for Biotechnology Information (NCBI) and registered.

### 2.14. Statistical Analysis

SPSS software (SPSS, Inc. No. 22) was used for statistical analyses. Categorical data were analyzed using either the Fisher exact test or the *χ*^2^ test. A significance level of *p* < 0.05 was deemed statistically significant.

## 3. Results

### 3.1. *P. aeruginosa* Isolation and *Antibiotic Sensitivity Pattern*

In total, 100 *P. aeruginosa* strains from clinical samples were obtained and identified through biochemical tests. According to the results, *P. aeruginosa* was isolated from respiratory samples (51%), urine (19%), blood (13%), ulcers and abscesses (12%), and others (5%), respectively (*p* < 0.001).

Analysis of the results using WHONET 5.6 software illustrated that out of 100 samples, 61% of samples were MDR that were selected for further evaluation. Based on antibiotic susceptibility pattern of MDR isolates, 98.3% of strains were resistant to ciprofloxacin, levofloxacin, and piperacillin-tazobactam (Table 1). Less than 10% of strains were intermediate to carbapenem family antibiotics (imipenem and meropenem), and more than 80% of strains were resistant to other antibiotics. Since metallo-beta-lactamase enzymes have a wide spectrum of enzymes that hydrolyze almost all beta-lactam classes, our results showed that among carbapenem-resistant isolates, 73.2% (41/56) of strains were positive for this enzyme.

### 3.2. Probiotic Isolation and Identification

A total number of 36 *Lactobacillus* strains were isolated from local dairy samples (milk and yogurt), and their antibacterial and antibiofilm effects against *P. aeruginosa* strains were investigated. Out of 36 *Lactobacillus* samples, the cell-free supernatants of two *Lactobacillus* strains (named P1 and P2) had an inhibitory effect (showed inhibition zones (on all *P. aeruginosa* strains. Both P1 and P2 strains were isolated from traditional buttermilk.

Additionally, bile and acid tolerance tests showed that two strains P1 and P2 were resistant to both bile and acid. The decrease in the number of *P. aeruginosa* through treatments with probiotics, after one and three hours, was less than 10^6^ bacteria, and the decrease in the number was not statistically significant (*p* > 0.05). Biochemical tests were performed to identify two *Lactobacillus* strains P1 and P2.

### 3.3. Identification of Selected Probiotic Bacteria, Sequence Analysis, and Registration in NCBI

Two strains of *Lactobacillus* P1 and P2, which had the highest inhibitory activity, were identified by phenotypic (biochemical tests) and genotypic (using the 16SrDNA method) tests. The PCR product was sequenced. The results of biochemical tests and blast sequence of 16SrDNA obtained in the sequence with the available data bank showed that both of the above bacteria were *L. rhamnosus* (B03 and D03 strains). Two genome sequences of bacteria were registered on the NCBI website with accession numbers OL451220 and OL451221.

### 3.4. Antimicrobial Effect of Probiotics against P. aeruginosa

The microtiter plate test in 24 wells confirmed the obtained results, which showed that with the simultaneous cultivation of the supernatant of two bacteria, P1 and P2 with *P. aeruginosa* bacteria, no change was observed in the light absorption of all bacteria. After 24 hours of incubation, no colonies were observed in the wells with concentrations of 1, 1/2, and 1/4, which indicates that the probiotic inhibited *P. aeruginosa* in these three concentrations, and in the concentrations of 1/8 and 1/16, the colony growth was observed.

Therefore, the concentration of 1/4 was considered as MIC. To determine MBC, 50 microliters of wells without turbidity, which included concentrations of 1, 1/2, and 1/4, was cultured on blood agar medium via the sterile swab and incubated at 37°C for 24 hours. The results showed no colony growth in the concentration of 1/4; consequently, the ratio of the concentration of MBC and MIC was similar. The result of the antibiofilm inhibitory effect of probiotics showed that the selected probiotic had antibiofilm activity and prevented the formation of *P. aeruginosa* strain biofilm in concentrations of 1, 1/2, and 1/4, but in the concentrations of 1/8 and 1/16, *P. aeruginosa* strains formed biofilm, and the probiotic strain could not inhibit them.

### 3.5. Antibiotic Sensitivity Pattern and Virulence Factor of P1 and P2 Strains

Determining the antibiotic susceptibility pattern with disc diffusion method for P1 and P2 strains showed that these probiotics were sensitive to most of the antibiotics including penicillin, ampicillin, linezolid, ciprofloxacin, erythromycin, clindamycin, and gentamicin and were resistant to vancomycin, teicoplanin, trimethoprim, sulfamethoxazole, and tetracycline.

To evaluate the virulence factors, DNase, amylase, beta-hemolysis, catalase, production of protease, and gelatinase enzymes tests were negative for both P1 and P2 strains.

### 3.6. Cytotoxicity

The cytotoxicity test against the L929 fibroblast cell line showed that the cytotoxicity was 7.3% and 6.6%, respectively, in the case of P1 and P2 bacteria, which was not significantly different from the control bacteria sample (5.6%) (*p* > 0.05) that indicated their low level of cytotoxicity.

Investigating the inhibitory effect of organic acids and determining their type and concentration by the HPLC method showed that by neutralizing the acidic pH of the liquid on two *L. rhamnosus* P1 and P2 strains, all *P. aeruginosa* strains grew.

**Figure 1:**
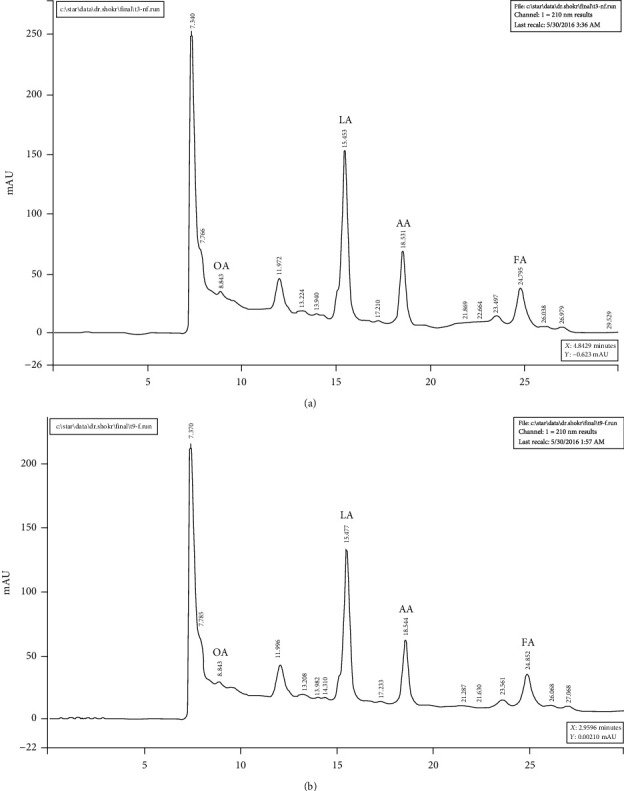
HPLC graphs of the analysis of the type and amount of organic acids in two *L. rhamnosus* P1 (a) and P2 (b) strains. OA: oxalic acid; LA: lactic acid; AA: acetic acid; FA: formic acid.

The two main organic acids according to their concentration were lactic acid and acetic acid, respectively. The concentration of lactic acid for P1 and P2 were 9.28 mL/L (87%) and 8.57 mL/L (87.5%), respectively; and the concentration of acetic acid for P1 and P2 was 0.93 mL/L (8.72%) and 0.86 mL/L (7.78%), respectively (Figure 1).

## 4. Discussion

The emergence and spread of antibiotic-resistant bacteria and antibiotic resistance genes are of concern to health care systems around the world [32–34]. In this study, the antibiotic resistance rate of *P. aeruginosa* strains causing nosocomial infections and the phenotypic abundance of metallo-beta-lactamase enzymes as a mechanism of carbapenem resistance in this bacterium were investigated [35]. On the other hand, *L. rhamnosus* strains were reported as potentially useful probiotic bacteria against these pathogenic strains. Similar to the previous studies, *P. aeruginosa* strains were frequently isolated from respiratory samples [36]. Ahmed et al. reported that most cases of isolated *P. aeruginosa* were detected in respiratory samples. This high frequency is related to the selective colonization of these strains causing infections in the respiratory system [37].

Our results are in agreement with other studies in Iran, where high resistance of *P. aeruginosa* strains to most antibiotics was found. This may be due to the higher rate of antibiotic administration in Iran without considering the antibiogram or antibiotic susceptibility analysis [38, 39]. Considering the importance of carbapenems in the treatment of resistant strains of *P. aeruginosa*, different frequencies of resistance to carbapenems have been reported [40]. The present study showed that less than 10% of *P. aeruginosa* strains were intermediate to carbapenems. Heidari et al. demonstrated that carbapenem resistance in *P. aeruginosa* is a problem in nosocomial infections and that resistance to carbapenem is significantly related to resistance to other antibiotics, exacerbating the condition and limiting treatment options [41].

Our results indicate that 74.5% of *P. aeruginosa* strains were positive for the metallo-beta-lactamase enzyme, which is a higher percentage compared to other regions. This discrepancy is likely due to variations in the frequency of these enzymes across different geographical regions and differences in antibiotic administration patterns, both of which can influence the prevalence of these enzymes. The amount of metallo-beta-lactamase enzymes in *P. aeruginosa* strains was previously reported 18%, 11%, and 42%, in Zahedan, Tehran, and Isfahan, respectively [14–16, 42]. Goel et al. reported in their study that the frequency of MBL enzyme in *P. aeruginosa* strains was about 54% in India [43].

The results of the present study showed that two selected *L. rhamnosus* exhibited a broad spectrum of activity and completely inhibited all *P. aeruginosa* strains examined. No growth was observed after simultaneous cultivation of P1 and P2 with *P. aeruginosa* strains, indicating their eradication in addition to growth inhibition and highlighting the potential ability of these probiotics. Probiotics with many therapeutic properties, especially with antimicrobial effects, can prevent the growth of pathogenic bacteria by producing various antimicrobial compounds such as lactic acids and lowering pH [18, 19]. In agreement with our study, the inhibitory effect of *Lactobacillus* strains against pathogenic bacteria such as *P. aeruginosa* has been reported [20–22].

Since biofilm is formed by probiotics, their beneficial antibacterial and anti-inflammatory properties play an important role in the host immune system [23, 24]. Our results showed that P1 and P2 were very strong biofilm formers, and this important property makes them potentially useful probiotics for industrial use. Moreover, the liquid on the surface of these two bacteria prevented biofilm formation in all *P. aeruginosa* strains, so biofilm formation was zero in the presence of the above probiotics. Similar studies have reported the preventive role of *Lactobacillus* in biofilm formation by pathogenic bacteria [25, 26]. Lenhard et al. reported that probiotics with antimicrobial and antibiofilm properties can be used to prevent and treat MRSA infections [27].

Probiotics must have some properties, such as resistance to bile and acid, production of antimicrobial substances, ability to bind to intestinal epithelial cells, and ability to accumulate themselves by preventing pathogen colonization, to be useful for treatment [28, 29]. Two selected *L. rhamnosus* strains can inhibit *P. aeruginosa* strains by exhibiting specific pathogen growth inhibition properties. The results showed that P1 and P2 were resistant only to vancomycin and teicoplanin, trimethoprim-sulfamethoxazole, and tetracycline and sensitive to the others. Since probiotics and antibiotics are used simultaneously in the treatment of some infectious diseases, if the probiotics are not resistant, it is the first organism that is destroyed [30]. Lactobacilli, the main bacteria used as probiotics, therefore possess intrinsic resistance to some antibiotics, making them suitable for coadministration with antibiotics. In their study, Wong et al. reported that all *Lactobacillus* strains in dietary supplements were resistant to vancomycin and teicoplanin [31].

In agreement with other studies, the *Lactobacillus* strains studied were negative in terms of catalase production, pathogenic DNase enzyme production, and pathogenic hemolysin factor production [32, 33]. The result of the cytotoxicity assay against the L929 fibroblast cell line was less than 8% for P1 and P2, confirming their safety. Different studies have reported different values for the cytotoxicity of probiotics. Shahid et al. reported a cytotoxicity rate of approximately 28%, which was higher than that of the present study [34], whereas other studies found a cytotoxicity rate of less than 10% [35, 36].

The results of the analysis of organic acids in the liquid of the two *Lactobacillus* strains by HPLC method showed that most of the organic acids in both samples were lactic acid and acetic acid. The strong inhibitory effect of these probiotics was most likely due to the high amount and presence of these two organic acids. The effect of organic acids produced by *Lactobacillus* strains, especially lactic acid and acetic acid, on the inhibition of pathogenic bacteria has been confirmed in many studies [37, 38]. Two broad-spectrum probiotics (P1 and P2) were effective against all pathogenic strains of *P. aeruginosa* and may represent a promising alternative to antibiotics.

## Figures and Tables

**Table 1:** The antibiotic resistance patterns of MDR-*P. aeruginosa* strains.

Antimicrobial category	Antibiotic	Resistant (%)	Intermediate (%)	Sensitive (%)
Aminoglycosides	Gentamicin	57 (93.5)	1 (1.6)	3 (4.9)
	Amikacin	52 (85.3)	3 (4.9)	6 (9.8)

Antipseudomonal cephalosporins	Ceftazidime	60 (98.4)	1 (1.6)	—
	Cefepime	54(88.7)	7 (11.3)	—

Antipseudomonal carbapenems	Meropenem	56 (91.8)	5 (8.2)	—
	Imipenem	56 (91.8)	5 (8.2)	—

Antipseudomonal fluoroquinolones	Ciprofloxacin	60 (98.3)	1 (1.8)	—
	Levofloxacin	60 (98.3)	1 (1.8)	—

Antipseudomonal penicillins+*β*-lactamase inhibitors	Piperacillin-tazobactam	60 (98.3)	1 (1.8)	—

## Data Availability

Data are available on request from the authors.
